# Co-designing Improved Communication of Newborn Bloodspot Screening Results to Parents: Mixed Methods Study

**DOI:** 10.2196/33485

**Published:** 2022-07-27

**Authors:** Jane Chudleigh, Lynette Shakespeare, Pru Holder, Holly Chinnery, Gemma Hack, Tanya Gill, Rachel Gould, Kevin W Southern, Ellinor K Olander, Stephen Morris, James R Bonham, Alan Simpson, Louise Moody

**Affiliations:** 1 Cicely Saunders Institute King’s College London London United Kingdom; 2 Pharmacy, Diagnostics and Genetics Sheffield Children's NHS Foundation Trust Sheffield United Kingdom; 3 Faculty of Sports, Health and Applied Science St Mary's University Twickenham United Kingdom; 4 Evelina London Children’s Hospital Guy's and St Thomas' NHS Foundation Trust London United Kingdom; 5 Inherited Metabolic Diseases and Newborn Screening Birmingham Women’s and Children’s NHS Trust Birmingham United Kingdom; 6 Department of Women's & Children's Health University of Liverpool Liverpool United Kingdom; 7 School of Health and Psychological Sciences City, University of London London United Kingdom; 8 Department of Public Health and Primary Care University of Cambridge Cambridge United Kingdom; 9 Institute of Psychiatry, Psychology and Neuroscience King's College London London United Kingdom; 10 Centre for Arts, Memory and Communities Coventry University Coventry United Kingdom

**Keywords:** experience-based co-design, neonatal screening, health communication, participatory research

## Abstract

**Background:**

Each year in England, almost 10,000 parents are informed of their child’s positive newborn bloodspot screening (NBS) results. This occurs approximately 2 to 8 weeks after birth depending on the condition. Communication of positive NBS results is a subtle and skillful task, demanding thought, preparation, and evidence to minimize potentially harmful negative sequelae. Evidence of variability in the content and the way the result is currently communicated has the potential to lead to increased parental anxiety and distress.

**Objective:**

This study focused on the development of co-designed interventions to improve the experiences of parents receiving positive NBS results for their children and enhance communication between health care professionals and parents.

**Methods:**

An experience-based co-design approach was used to explore experiences and co-design solutions with 17 health professionals employed in 3 National Health Service Trusts in England and 21 parents (13/21, 62% mothers and 8/21, 38% fathers) of 14 children recruited from the same 3 National Health Service Trusts. Experiences with existing services were gathered via semistructured interviews with health professionals. Filmed narrative interviews with parents were developed into a composite film. The co-design process identified priorities for improving communication of positive NBS results through separate parent and health professional feedback events followed by joint feedback events. In total, 4 interventions were then co-designed between the participants through a web-based platform.

**Results:**

Parents and health professionals provided positive feedback regarding the process of gathering experiences and identifying priorities. Themes identified from the parent interviews included impact of initial communication, parental reactions, attending the first clinic appointment, impact of health professionals’ communication strategies and skills, impact of diagnosis on family and friends, improvements to the communication of positive NBS results, and parents’ views on NBS. Themes identified from the health professional interviews included communication between health professionals, process of communicating with the family, parent- and family-centered care, and availability of resources and challenges to effective communication. In response to these themes, 4 interventions were co-designed: changes to the NBS card; standardized laboratory proformas; standardized communication checklists; and an email or letter for providing reliable, up-to-date, condition-specific information for parents following the communication of positive NBS results.

**Conclusions:**

Parents and health professionals were able to successfully work together to identify priorities and develop co-designed interventions to improve communication of positive NBS results to parents. The resulting co-designed interventions address communication at different stages of the communication pathway to improve the experiences of parents receiving positive NBS results for their children.

**International Registered Report Identifier (IRRID):**

RR2-10.1186/s40814-019-0487-5

## Introduction

### Background

Newborn bloodspot screening (NBS) in England involves collecting a small sample of blood on a special card from a baby’s heel on day 5 of their life. This is then sent to an NBS laboratory to be analyzed. Positive NBS results are reported to relevant clinical teams, often using locally developed proformas [1] who then communicate the result to the family. Each year in England, almost 10,000 parents are informed of their children’s positive NBS results approximately 2 to 8 weeks after birth depending on the condition [2,3]. The purpose of NBS is to identify presymptomatic babies affected by one of 9 conditions currently screened for to enable treatment to be initiated early to improve outcomes for the child. The conditions are sickle cell disease (SCD), cystic fibrosis (CF), congenital hypothyroidism, phenylketonuria, medium-chain acyl-CoA dehydrogenase deficiency, maple syrup urine disease (MSUD), isovaleric acidemia, glutaric aciduria type 1, and homocystinuria (pyridoxine-unresponsive)—the latter 6 collectively referred to as inherited metabolic diseases. The clinical spectrum in screen-positive cases varies enormously and, consequently, the message to parents needs to be carefully crafted to prepare for a range of outcomes.

Communication of positive NBS results is a subtle and skillful task that demands thought, preparation, and evidence to minimize potentially harmful negative sequelae [4-8]. For instance, the perceived lack of knowledge of the person communicating the NBS result rather than the actual result has been linked to parental distress [4]. Poor or inappropriate communication strategies for positive NBS results can also influence parental outcomes in the short term [4-7,9,10] but may also have a longer-term impact on children and families [8]. Evidence suggests that the distress caused can manifest in several ways, including arguments between couples, apportioning of blame [4,6,11], alteration of life plans and inability to conduct tasks of daily living such as going to work or socializing [4], long-term alterations in parent-child relationships [8], and mistrust and lack of confidence affecting ongoing relationships with health care staff [6]. There is also evidence of increased parental concern resulting in parents reducing their child’s interaction with others, particularly in the case of CF [4]. Parents also experience poor intra- and interpersonal relationships within their family systems and more widely [12].

This supports the importance of ensuring that the initial communication of positive NBS results is handled sensitively and considers individual parent characteristics to minimize parental distress and the consequences of this distress, as well as the knowledge and experience of the person imparting the result. The choice of approach is, to some extent, influenced by the seriousness of the condition identified and the need for an immediate or less immediate response. In one study, parents who had received the screening results from a CF specialist were more satisfied than those who had received the screening results from the maternity ward [13]. In another study, information received by telephone was less satisfactory to parents of children diagnosed with CF (odds ratio 2.23; *P*=.04) or parents of younger infants (odds ratio 0.93 per day older; *P*=.001) [10]. Results delivered over the phone by staff not known to the families or without condition-specific knowledge were viewed less favorably and contributed to parental dissatisfaction, anxiety, and distress [9].

Recognizing the need to work with parents and health professionals to improve this communication, the *Rethinking Strategies for Positive Newborn Bloodspot Screening Result Delivery: a process evaluation of co-designed interventions* project sought to develop, implement, and evaluate new interventions to improve the delivery of initial positive NBS results to parents. This mixed methods study comprised 3 main phases. Phase 1 involved a national survey using telephone interviews to explore current approaches to the communication of positive NBS results [14] and inform the selection of 2 study sites for the remaining phases. The second phase used the principles of experience-based co-design (EBCD) to explore health professionals’ and parents’ experiences of delivering and receiving positive NBS results, respectively. Findings from interviews with health professionals have been published elsewhere [1]; sections of this paper related specifically to these findings have been reproduced from *BMJ Open* under license CC-BY-4.0. In addition, EBCD was used to develop interventions for communicating positive NBS results to parents. In phase 3, the interventions were evaluated in 2 selected case study sites (2 NBS laboratories that served 3 National Health Service [NHS] Trusts in England) [15].

### Aim

The aim of the research reported in this paper was to describe the use of a modified version of EBCD during phase 2 to develop co-designed interventions to improve the experiences of parents receiving positive NBS results for their children and enhance the communication between health care professionals and parents.

## Methods

### Overview

This formative study was underpinned by family systems theory (FST) [16] because of the potential vulnerability of family relationships if the initial positive NBS result information is not shared as effectively and empathetically as possible [17]. In FST, all components of the family are regarded as interdependent—what happens to one member will affect all other members of the family directly and indirectly. FST postulates that family functioning has the potential to be affected by an event such as the communication of the initial positive NBS result and, subsequently, facilitating the coping mechanisms used and the adaptation of families to the NBS result is paramount.

The co-design process was informed by the EBCD toolkit [18]. EBCD was selected because of its focus on service users and health professionals working in partnership to develop and improve health services. This was felt to be particularly appropriate as family-centered care, which includes working in partnership with the family, is the principal philosophy of pediatric care in many countries worldwide [19]. EBCD is an approach to improving health care services that draws on participatory design and user experience to bring about quality improvements in health care organizations [20]. This involves focusing on and designing patient or carer experiences rather than just systems and processes [21-23]. The co-design process enables staff, patients, and carers to reflect on their shared experiences of a service and then work together to identify improvement priorities, devise and implement changes, and then jointly reflect on their achievements. EBCD was piloted in an English head and neck cancer service in 2005 [21]. After a subsequent project in an integrated cancer unit, a web-based toolkit [18] was developed as a free guide to implement the approach. A recent systematic review identified 20 studies that had used EBCD mainly in mental health and cancer services in the United Kingdom. This review highlighted variations in the use of EBCD, with many of the studies eliminating or modifying some of the EBCD stages. It has been recognized that the disadvantages of EBCD include it being time-consuming and expensive. Until recently, EBCD had mainly been used with adult service users or their carers or family members. The use of EBCD with parents with or without the participation of their children is still quite novel, having only been explored more recently and with adaptations to the process [24-26]. Therefore, this study also builds on knowledge of using this method with parents.

**Figure 1:**
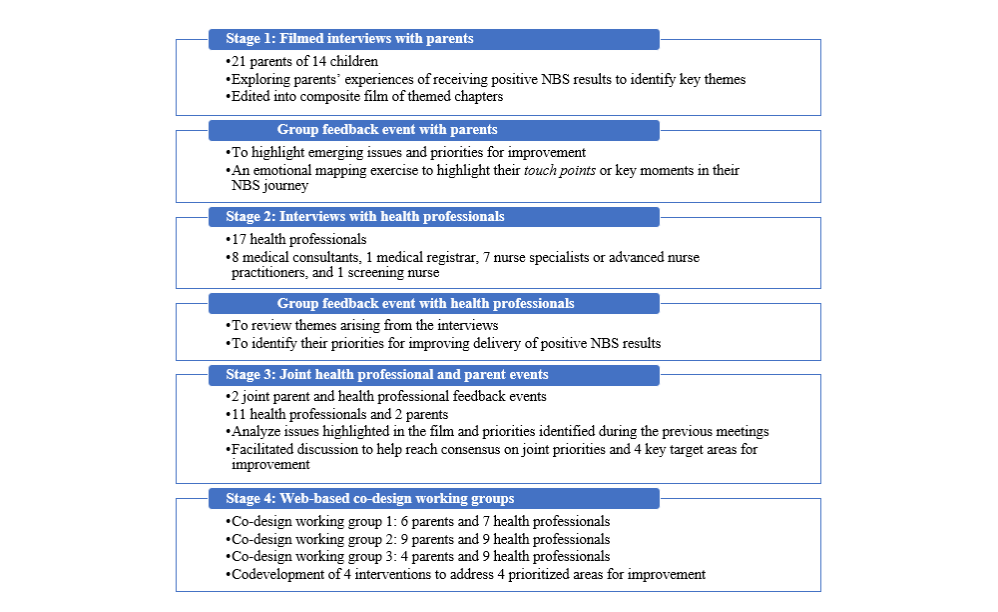
Adapted experience-based co-design approach. NBS: newborn bloodspot screening.

The EBCD process was modified to gather parents’ and health professionals’ experiences and agree on areas for improvement in the communication of positive NBS results to families. It followed four stages (Figure 1): (1) engaging health professionals and gathering experiences (the findings from health professional interviews have been published elsewhere [1]), (2) engaging parents and gathering their experiences, (3) bringing parents and health professionals together to share experiences and identify priorities for improvement, and (4) web-based co-design activities.

### Patient and Public Involvement

Patient and Public Involvement (PPI) was instrumental in the design and conduction of this study. A total of 8 parents of babies who had received a positive NBS result for one of the 9 screened conditions formed a PPI group that met every 6 months for the duration of the study. Their suggestions were incorporated into the study design, data collection tools, and data analysis and dissemination. The PPI group was presented with data from the annual reports of the NBS programs and made suggestions as to which sites should be used during the co-design process. In addition, views of representatives from charities for the screened conditions, including Metabolic Support UK, the British Thyroid Foundation, the CF Trust, and the Sickle Cell Society, were also incorporated.

### Study Sites and Sampling

#### Overview

The study sites consisted of 3 NHS provider organizations (Trusts) in England served by 2 NBS laboratories (study sites) that process comparable numbers of positive NBS reports annually for each of the 9 conditions currently included in the NBS program. These consisted of 2 Trusts in Greater London served by 1 NBS laboratory and 1 NBS laboratory in the West Midlands that processed 128 and 129 positive NBS results, respectively, in 2017 to 2018.

Informed by previous successful EBCD projects [20,22,27], we recruited a purposeful sample of parents across the 2 study sites. This ensured the participation of parents who (1) had received a positive NBS result for their child (2) in the previous 3 to 36 months, as well as ensuring (3) the representation of all screened conditions. Parents were identified as potential participants by health professionals communicating positive NBS results. During a routine hospital appointment, health professionals asked parents if they would be willing to talk to a member of the research team about the study. If the parents agreed, a member of the research team met with them, explained the study, and provided a participant information sheet. Parents were asked if they would be willing to share their contact details so that a member of the research team could contact them the following week to answer any questions they might have about the study. During the follow-up contact, if parents were agreeable, an appointment was made to undertake the filmed interview at a convenient time and location of the parents choosing (all parents chose to be interviewed at home).

A 2-stage sampling approach was used to recruit health professionals involved in communicating positive NBS results in the preceding 6 months at the 2 study sites. Participants were first sampled purposively based on their experience with reporting or communicating positive NBS results, followed by a second stage of snowball sampling. Members of relevant clinical teams (medical consultants, general pediatricians, nurse specialists, and specialist screening nurses) were initially identified through individual Trust websites, contacted via email, and invited to participate. If no response was received, a follow-up email was sent after 1 week. Health professionals who responded were asked if there were any other members of their clinical teams that the research team should contact to ensure that the views were representative.

The sample sizes for both parents and health professionals were influenced by previous EBCD projects and the EBCD toolkit [18].

#### Stage 1: Engaging Parents and Gathering Experiences

##### Participants

Filmed interviews were conducted with 21 parents: 13 (62%) mothers and 8 (38%) fathers of 14 children recruited from 3 NHS Trusts in England served by 2 NBS laboratories. Of the 21 parents, 18 (86%) identified as White British, 1 (5%) identified as White European, 1 (5%) identified as Asian British, and 1 (5%) identified as Black British. Their ages ranged from 25 to 44 (median 37) years. Of the 14 children, 4 (29%) had CF; 3 (21%) had medium-chain acyl-CoA dehydrogenase deficiency; 2 (14%) had phenylketonuria; 1 (7%) had MSUD; 1 (7%) had congenital hypothyroidism; 1 (7%) had SCD; 1 (7%) had been designated CF screen-positive, inconclusive diagnosis; and 1 (7%) had received a false positive result for CF. Of the 14 children, 7 (50%) had older siblings, only one of whom had also been diagnosed with a condition (CF) via NBS. A total of 14% (2/14) of the children were twins (both had CF), and 36% (5/14) did not have any siblings. At the time of the interview, the ages of the children ranged from 10 to 107 (median 43) weeks.

##### Data Collection

We conducted filmed narrative interviews with parents across the 2 study sites between September 2018 and March 2019 exploring parents’ experiences of receiving positive NBS results to identify key themes (touch points). Interview questions were guided by the principles of FST [16,17] and focused on the impact of receiving a positive NBS result on the family and on their relationships with each other, with their children, and also with their wider support network, including their friends. The interviews lasted between 14.5 and 47.4 (median 26.4) minutes. Parents were asked to talk about their experience of receiving their child’s positive NBS result both in terms of the process and any emotions or feelings this had caused and why.

##### Data Analysis

FST [16,17] informed the development of themes identified from parent interviews. This included consideration of parent reactions to receiving the positive NBS result and of how this had affected them as parents, individuals, and partners, as well as the impact of the diagnosis on family and friends, reflecting the tenets of holism and interdependence that are fundamental to FST. Themes identified from parent interviews were developed into a composite film in April 2019. The film was used to capture parents’ experiences of receiving their children’s positive NBS results and provide rich information to guide the development of the co-designed interventions.

Following the interviews, parents at each study site were invited to a parent feedback event (1 in the West Midlands and 1 in London) to enable them to watch the composite film and discuss key priorities to improve the communication of positive NBS results to families. These events were guided by the web-based EBCD toolkit [18] and accompanying web-based resources, including the invitation, agenda, and feedback templates. Parents were invited to view the composite film of the interviews to ensure that it was a fair and valid representation of their shared experiences. This was used to inform a facilitated group discussion that lasted approximately 3 hours to highlight emerging issues and priorities for improvement. In addition, an emotional mapping exercise was conducted to highlight their *touch points*, or emotionally charged or key moments in their NBS journey. During this discussion, parents were asked to work together to consider 4 key questions (Textbox 1).

Touch points were gathered from the composite film and the emotional mapping exercise to highlight priorities to share with health professionals.

**Prompts for the parent feedback event.**

**Key questions**

- Do you feel the film represents your views and experiences?
- What parts of your journey were you happy with? Why?
- What parts of your journey do you think could be improved? How?
- What questions would you like to ask health professionals?

#### Stage 2: Engaging Health Professionals and Gathering Experiences

##### Participants

Health professionals were recruited from the same 3 NHS Trusts in England served by 2 NBS laboratories. In total, 20 health professionals involved in communicating positive NBS results in the preceding 6 months were emailed and invited to participate, of whom 2 (10%) did not respond to the invitation and 1 (5%) did not communicate the initial positive screening result and was therefore ineligible. In line with the EBCD approach [18], 16 face-to-face interviews were conducted with 17 health professionals (2/17, 12% requested to be interviewed together), of whom 8 (47%) were from one of the NBS laboratories and the remaining 9 (53%) were split across the 2 Greater London Trusts served by the other NBS laboratory. Participants with experience with all 9 screened conditions were included. The sample consisted of 47% (8/17) medical consultants, 6% (1/17) medical registrars, 41% (7/17) nurse specialists or advanced nurse practitioners, and 6% (1/17) screening nurses. The length of experience with NBS ranged from 2 to 38 (median 8) years. The interviews lasted 37 minutes on average (SD 10.51, range 19-58 minutes) [1].

##### Data Collection

Semistructured telephone interviews comprising closed- and open-ended questions were conducted between September 2018 and February 2019 to identify the approaches used to communicate positive NBS results from NBS laboratories to health professionals. Data collected included the mode of communication strategy (face-to-face, letter, telephone, or email), the resources involved in each communication strategy, who provided the information and their role, and the location (colocated or alternative site) of relevant services for each condition.

**Figure 2:**
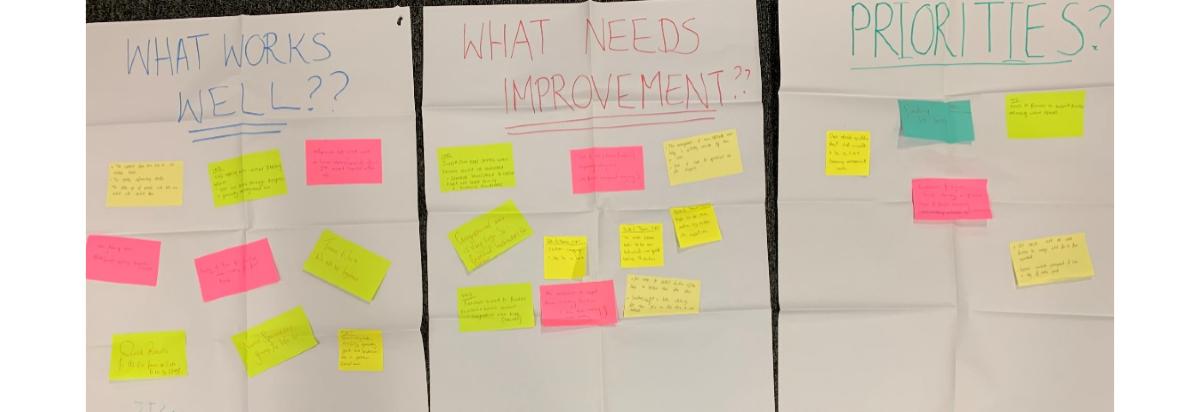
Illustrative flip charts from health professional workshops.

After the interviews, health professionals at each site were invited to attend a health professionals’ event to review themes arising from the interviews and identify their priorities for improving the delivery of positive NBS results (1 in the West Midlands and 2 in London). These events were guided by the web-based EBCD toolkit [18] and the accompanying web-based resources, including the invitation and agenda template. The findings of the health professional interviews were presented via a Microsoft PowerPoint presentation using direct quotes to illustrate the points made. The participants were encouraged to reflect on what they considered to be working well, what they thought required improvement and, from this, key priorities to improve the communication of positive NBS results to families. Health professionals were asked to record their thoughts on a flip chart paper so it could be shared with the whole group (Figure 2).

##### Data Analysis

The interviews were analyzed thematically; an inductive method of data analysis was used, and themes were generated using a latent approach. This provided a deeper understanding of the approaches used to communicate positive NBS results to families [28]. In total, 2 members of the research team (JC and HC) coded 1 interview transcript separately. These codes were then compared to inform and align code development [29], and a codebook was developed [30]. A further 4 transcripts were then coded separately by the same 2 members of the research team using the codebook. These separately coded transcripts were then compared; intercoder reliability was 84%. Following this, the same 2 members of the research team coded the remainder of the transcripts using the codebook. Once this initial coding had been completed, data for each code were compared to ensure consistency in coding and to enable the codes to be collapsed into themes. All quotes for each theme were collated to inform theme development. This was an ongoing, iterative process; new codes were developed, and the definition of codes was refined as the analysis progressed [1].

#### Stage 3: Bringing Health Professionals and Parents Together

##### Participants

Health professionals and parents who had taken part in the previous events were invited to take part in one of 2 joint parent-health professional feedback events: 1 in the West Midlands and 1 in London. A total of 6 health professionals and 1 parent joined the event in the West Midlands, and 5 health professionals and 1 parent joined the London event.

##### Data Collection

Mixed health professional and parent events [31] were held at each of the study sites. These events were face-to-face and took approximately 2 to 3 hours. These events were guided by the web-based EBCD toolkit [18] and the accompanying web-based resources, including the invitation and agenda template. During these events, a parent representative (discussed and agreed upon before the meeting) was invited to introduce and share the composite film with health professionals. An unstructured discussion followed to analyze issues highlighted in the film and priorities identified during the separate health professional and parent meetings. This was followed by a facilitated discussion to help reach a consensus on joint priorities. In total, 4 key target areas for improving the delivery of positive NBS results [20,27,32] were agreed upon to be the focus of the co-design activities over the following 8 weeks (July 2019 and August 2019).

##### Data Analysis

During the joint health professional and parent feedback event, the participants were asked to write on Post-it notes placed on flip chart paper what they currently considered to be working well, what areas they thought needed improvement, and priorities. These were shared with the group and, following a facilitated group discussion, shared priorities were agreed upon, and key target areas were identified for improvement of communication of positive NBS results to parents.

#### Stage 4: Co-design Working Groups

##### Participants

A total of 3 co-design working groups were run, each attended by 12 to 18 participants (Figure 1). The participants were permitted to be part of more than one co-design working group if they wished.

##### Data Collection

The co-design working groups took place in July 2019 and August 2019. EBCD is typically undertaken through face-to-face events [18]. It was modified in this case as health professionals and parents requested that the co-design working groups be held on the web. The rationale for this was to offer more flexibility to share resources but also to facilitate communication and negotiation between health professionals and parents regarding the proposed co-designed interventions.

The web-based platform Basecamp [33] was used to host the web-based co-design working groups. Each co-design working group was set up as a different group; those who had indicated that they would be interested in a particular co-design working group were invited via email to participate.

Ground rules were jointly agreed upon at the outset and posted on the web. The *Message Board* was used to invite participants (a mixture of health professionals and parents in each co-design working group) and remind them of the purpose of the groups. The composite film as well as Microsoft PowerPoint presentations and priorities from the separate and joint parent and health professional events were made available. Example interventions based on discussions held during the separate and joint parent and health professional feedback events were also shared, and members of the co-design working groups were asked to provide feedback and comments. The *Campfire* function was used for discussion related to iterations of all documents. Each time new documents were uploaded, a message was sent to the members of the relevant co-design working group via the *To-dos* function.

**Figure 3:**
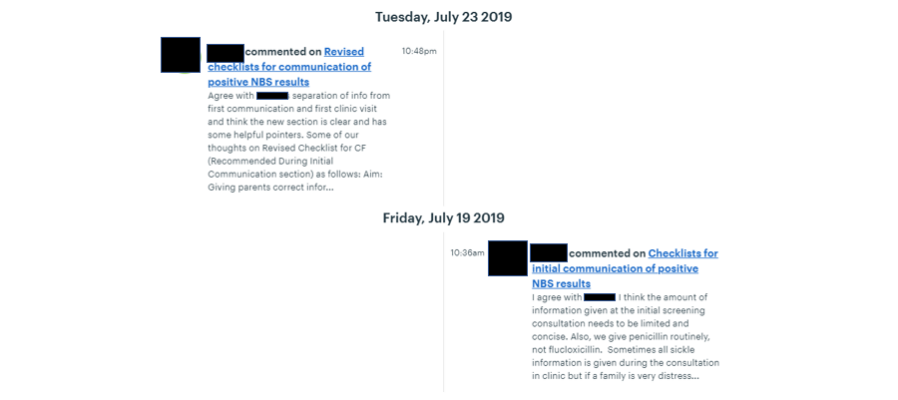
Redacted example of communication during the co-design working groups.

The participants were asked, over a period of 8 weeks from July 2019 to August 2019, to post comments on documents and files that were uploaded. Members of each group were sent a message approximately weekly or when new or revised documentation was uploaded to the web-based portal asking them to review the information and provide feedback. They also used the web-based discussion board to communicate with each other and develop the co-designed interventions. An example of communication between parents and health professionals through this platform is shown in Figure 3. Versions of relevant documents were updated in light of health professionals’ and parents’ comments until a consensus was reached regarding the suitability of the proposed interventions. Both parents and health professionals engaged effectively with the web-based co-design working groups. Comments and feedback were left at all times of the day and night, indicating that using the web-based forum enabled participants to contribute to the co-design working groups at times that were convenient for them. Conducting the co-design working groups on the web also appeared to mitigate any potential imbalance in terms of perceived power hierarchies between parents and health professionals [34], with both contributing and replying to each other’s comments. Furthermore, being able to monitor which participants had contributed comments or feedback meant that it was easier to direct questions to participants who had been less forthcoming in discussions and encourage their involvement in a nonconfrontational manner.

### Ethics Approval

All potential participants were given the choice to take part or not and were reminded of their right to withdraw from the study at any time. Written informed consent was obtained from all participants. This study is part of a larger program of work [35] and was approved by the London – Stanmore Research Ethics Committee (17/LO/2102).

## Results

### Experiences and Views

A total of 5 themes were identified from the interviews with health professionals: communication between health professionals, process of communicating with the family, parent- and family-centered care, and availability of resources and challenges to effective communication. Data from the interviews with health professionals have been published in full elsewhere [1].

Themes identified from the interviews with parents included impact of initial communication, parental reactions, attending the first clinic appointment, impact of health professionals’ communication strategies and skills, impact of diagnosis on family and friends, improvements to the communication of positive NBS results, and parents’ views on NBS. The findings were presented as a composite film (available via the study blog [36]) to capture and illustrate parents’ experiences of receiving their children’s positive NBS results and provide rich information to guide the co-design activities. The film is presented in 7 sections that reflect the stages of parental experiences and their journeys through screening. The common experiences or *touch points* for parents that were reflected in each section of the film are summarized in Textbox 2.

**Touch points from the composite film.**

**Section 1: initial communication**

- Various methods of communication were used including face-to-face, telephone, and SMS text message.
- The characteristics of the person communicating the newborn bloodspot screening (NBS) result were important.
- The person communicating the NBS result was not always knowledgeable about the condition and could be viewed as unreliable.
- Mothers frequently communicated the result to their partners.
- The NBS result was perceived to be delivered as “bad news,” which contributed to their initial feelings of fear and pain (see below).

**Section 2: parents’ reactions**

- Common feelings: shock, fear, confusion, pain, and disbelief
- The positive NBS result was traumatic, upsetting, and devastating.

**Section 3: attending the first clinic appointment**

- The wait between the initial communication and the first clinic appointment was difficult (this was normally <24 hours).
- Practical arrangements had to be made at short notice (eg, travel, which could be expensive, and childcare for other children).
- The initial clinic appointment was exhausting.

**Section 4: health professionals’ communication**

- Condition-specific specialists were found to be positive, supportive, knowledgeable, empathetic, reassuring, and credible.

**Section 5: impact of diagnosis on family and friends**

- Some parents reported that the positive NBS result had brought them closer together.
- Some felt it had created a strain on their relationship.
- Some felt it had affected their relationship with their baby in terms of bonding and attachment.
- Parents felt responsible for telling family and friends.

**Section 6: improvements to the communication of positive NBS results**

- Those involved should be knowledgeable about the conditions and the process when communicating positive NBS results.
- Partners should be informed at the same time as mothers.
- An SMS text message alert (or similar) could help prepare parents to receive the positive NBS result.
- The NBS result should be communicated to parents by a condition-specific specialist.
- Information should be provided immediately after the child’s positive NBS result is relayed.

**Section 7: parents’ views on NBS**

- The NBS program was viewed very favorably.
- New parents should be encouraged to participate in the NBS program.
- Midwives should be familiar with the conditions included in NBS.

### Priorities for Improving Communication

During a facilitated discussion after watching the film of parental experiences, feedback from parents and health professionals was narrowed down to a short list of priorities for them to explore together to improve communication. These are summarized in Table 1.

**Table 1:** Summary of participants’ priorities to improve communication.

Category	Parents’ priorities	Health professionals’ priorities
Changes to NBS^a^ card	How the parent would like to be contacted Significant other’s contact details on the card (as well as the mother’s) Whether a translator is needed Email address of the parents	Inclusion of a question on the NBS card asking the parents how they would like to be contacted: Skype, telephone, or email Addition of a parent’s email address to the NBS card
Initial communication	Being told by the same person they will see at the first clinic appointment If parents are given their child’s result over the telephone, care should be coordinated so that they can speak to a health visitor (registered nurses or midwives who have undertaken additional training and work mainly with children from birth to 5 years and their families) or midwife after for support (they do not need to have knowledge of the condition) Parents to be told who they can or should bring to the first clinic appointment	Templates for communication to clinical teams and initial communication to families that should be condition specific Information for families about who should attend the initial clinic appointment
Follow-up communication	Parents to be emailed details of the first clinic appointment Information for family and friends Being signposted at this stage with trustworthy and reliable resources or websites	Following delivery of the positive NBS result by phone, email parents with appointment letter, directions, and condition-specific leaflet; this can be done by administrators or the CNS^b^ Information resources for families and extended families
Service provision	Financial support for families to attend the initial clinic appointment	A centralized system for CHT^c^ Formulation of diagnostic services especially out of hours (so laboratories can conduct confirmatory testing over the weekend) Financial support for families to attend the initial clinic appointment

^a^NBS: newborn bloodspot screening.

^b^CNS: clinical nurse specialist.

^c^CHT: congenital hypothyroidism.

### Co-design Working Groups and Interventions

During the joint parent and health professional groups, the participants narrowed down the initial priorities (Table 1). Through discussions and shared expertise on the potential causes of communication issues, they decided on the focus of each of the co-design working groups. This is summarized in Table 2.

The participants agreed that changes to the NBS card (completed during the heel prick test by the midwife) were required to address the challenge of having all the information necessary to contact the family (1) in a timely (condition-specific) manner and (2) according to parental preferences.

There was also a focus on standardized laboratory proformas for use in the NBS laboratories. This focus emerged from a need for consistent and thorough information to be relayed to clinical teams to facilitate making contact with the child’s family following a positive NBS result.

Parents recognized inconsistent communication approaches. It was agreed that standardized communication checklists for health care professionals would guide conversations throughout the screening journey and support health professionals with less condition-specific knowledge or experience.

A template email or letter to the parents was proposed as the fourth intervention. This would be sent by the clinical team after the initial communication with the parents. The purpose would be to provide reliable up-to-date, condition-specific information for parents following the communication of the positive NBS result.

Through the co-design process, ideas and documentation were reviewed and iterated through the Basecamp platform until a consensus was reached regarding the suitability of the proposed interventions. Overall, there were 6 iterations of the NBS card, 5 iterations of the laboratory proformas, 8 iterations of the communication checklists, and 6 iterations of the email or letter for providing information to parents following the communication of the positive NBS result. Examples of the final versions are outlined in the following sections.

**Table 2:** Co-design working groups (CDWGs).

Group	Proposed intervention	Need
CDWG 1	Changes to the NBS^a^ card completed during the heel prick test by the midwife Standardized laboratory proformas for use in the NBS laboratories	To ensure that health professionals have all the required information to make rapid contact and that parents are contacted in their preferred way To ensure that the required information is consistently transferred from the laboratories to clinical teams
CDWG 2	Standardized communication checklists for health care professionals	To ensure that the required information is relayed consistently to families during the initial communication
CDWG 3	A template email or letter to parents	To provide reliable, up-to-date, condition-specific information for parents following the communication of the positive NBS result

^a^NBS: newborn bloodspot screening.

### The NBS Card

**Figure 4:**
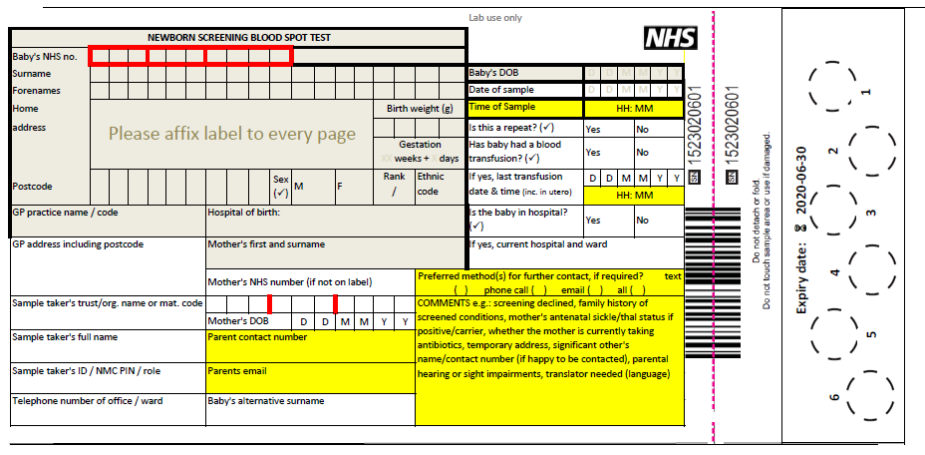
New newborn bloodspot screening card.

The final version of the proposed NBS card included the addition of parents’ preferred method of contact. This aimed to prompt conversation between midwives and parents at the time the NBS sample was taken regarding the possibility of them being contacted in the future if the results were positive as well as to ensure that parents were involved in the decision about how they might be contacted. Alternative contact details of a significant other were also added to act as a second line of contact should a clinician be unable to reach the mother following the NBS result. The parents’ email addresses were added to aid future communication and contact. Finally, the option to add information related to any hearing or sight impairments or language needs that might hinder future communication with parents was added to the NBS card. The changes and additions are shown in Figure 4.

### Standard Laboratory Proformas

**Figure 5:**
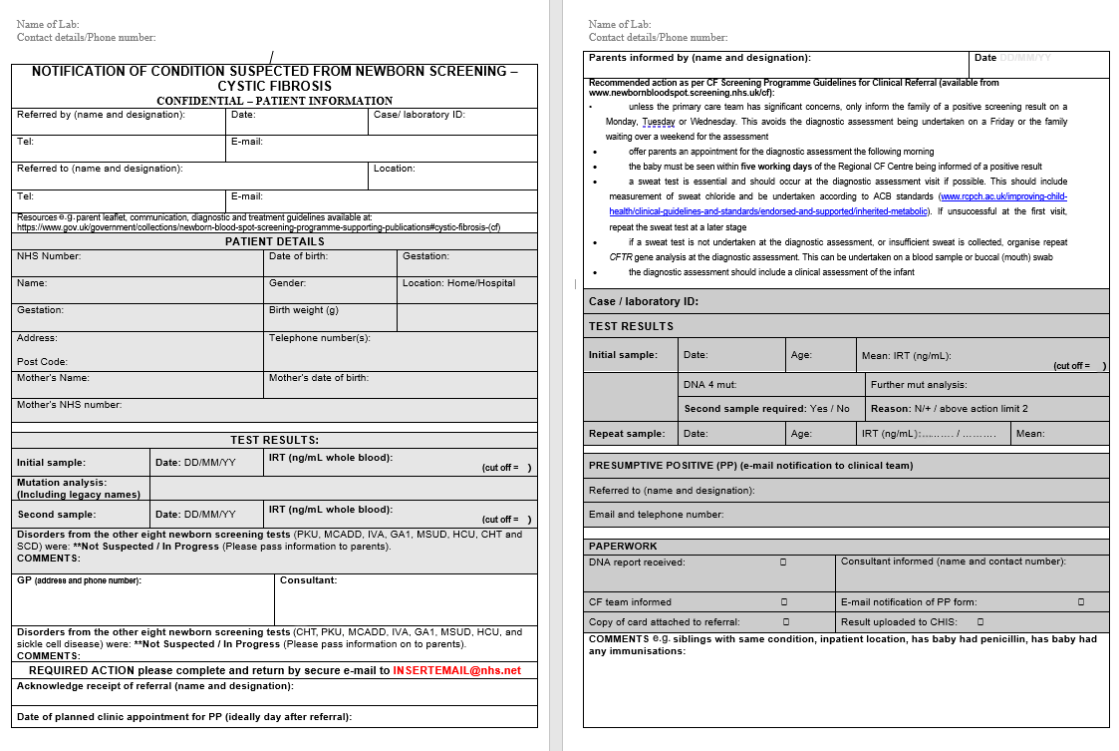
Example co-designed laboratory proforma for cystic fibrosis.

The standard laboratory proformas built on those developed by the Department of Clinical Chemistry and Newborn Screening at the Sheffield Children’s NHS Foundation Trust. The proformas were condition specific and included a front page that was mainly intended for completion by the NBS laboratory and a section for completion by the clinicians to be fed back to the NBS laboratory. On the reverse side, there was a reminder of the current referral guidelines, more information about the child’s NBS result, and a checklist focused on steps in the referral process. Additions as a result of the co-design process included information related to recommended actions following a positive NBS result for each condition and a comment section to allow clinicians to record suggested condition-specific relevant information (Figure 5).

### Communication Checklist

**Figure 6:**
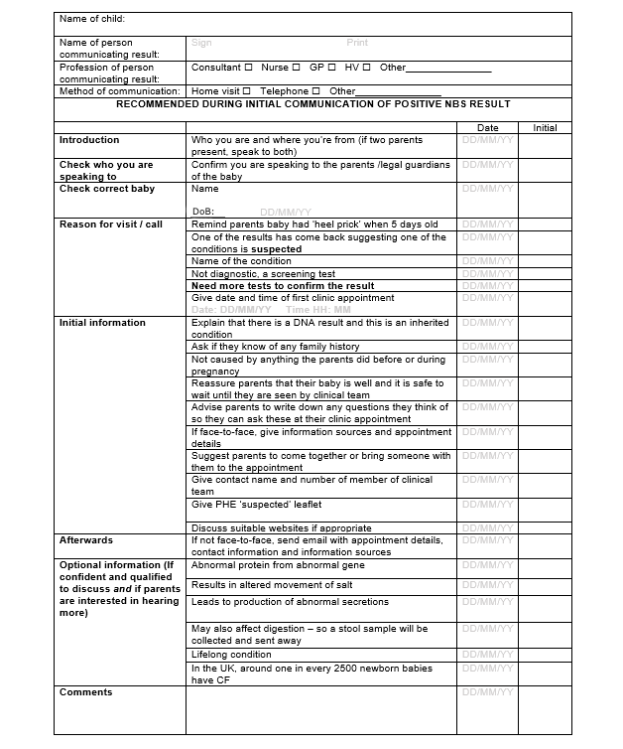
Example communication checklist for a child with suspected cystic fibrosis by newborn bloodspot screening.

The communication checklists were initially intended to focus on the initial communication of the positive NBS result. However, during the co-design working groups, the participants indicated that they would like the checklists for each stage of the families’ NBS journey to include the initial communication (Figure 6), the initial clinic visit, and subsequent clinic visits. It was thought that this would enable all information about the child and family’s NBS journey to be recorded in one place. This would also act as an aide-mémoire for subsequent clinicians when seeing the child and family and mitigate the need for parents to recount their story to different clinicians. The initial communication checklists were built on those developed by the CF teams at Sheffield Children’s Hospital and King’s College Hospital and the Newborn Screening Team at Birmingham Children’s Hospital to include more detailed condition-specific information as well as optional information that could be included if deemed appropriate. The checklists for subsequent clinic visits were developed with clinical teams and parents during the co-design process.

### Email or Letter Template

The email or letter template was intended to be sent to parents immediately after the initial communication of the positive NBS result. These built on those developed by the pediatric metabolic clinical nurse specialists at St Thomas Hospital. The purpose was to congratulate parents on the birth of their baby, reiterate why they had been contacted about the NBS, and provide details regarding what would happen next, including details of when and where they needed to take their baby for confirmatory testing. It was also recommended that reliable condition-specific links to information sources be included. The text was drafted and revised with input from the co-design working group until they agreed that the language and style of communication were appropriate and all information for all 9 conditions currently screened for was included.

### EBCD Process

The participants were asked to reflect and provide feedback on their experience of the EBCD process using the template provided by the EBCD toolkit [18]. This included a 5-point Likert-type scale ranging from *excellent* to *very poor*. All parents (21/21, 100%) rated viewing the composite film of parents’ experiences as *excellent*, their experience of being filmed as *good* or *excellent*, meeting other parents and talking about their experiences as *excellent*, and the emotional mapping exercise as *good* or *excellent*. They felt that the priorities agreed upon at the end of the parent event reflected their own experiences of what needed to be improved. A total of 29% (5/17) of the health professionals provided feedback and indicated that their overall impression of the health professional feedback event was *excellent* and an *excellent* way to reflect on experiences at work.

## Discussion

Uncertainty has been described as the single common challenge faced by patients who receive health care and the health professionals who provide it [37]. NBS by definition is not diagnostic and, as such, uncertainty in terms of clinical and prognostic outcomes is inevitable when communicating the initial NBS result [38]. In this study, parents and health professionals were able to successfully work together to identify priorities and develop co-designed interventions to improve the communication of positive NBS results using a modified EBCD approach.

### Parents’ Experiences of Receiving NBS Results

Consistent with previous research [9,10,13,39-42], parents in this study reported receiving NBS results in a range of ways, including face-to-face and via telephone and SMS text message, from a variety of clinicians, including nurses, physicians, and health visitors. The method used is, to some extent, influenced by the seriousness of the condition identified and the need for an immediate or less immediate response. MSUD and sickle cell carrier status would, for instance, be expected to be treated very differently in relation to the approach adopted. Furthermore, the content of the communication was less well defined and was, to some extent, determined by the person delivering the result. Current UK guidance states that the health professional delivering the news should be “appropriately trained” [43,44]. This is important as, similar to previous research [4,9,13,39,45], knowledge of the person communicating the result was considered important in this study to provide reassurance and allay parental fears.

In addition, parents in this study expressed the importance of the personal and professional attributes of the person delivering the news. In terms of personal attributes, this included being kind, empathetic, and supportive (physically and verbally) and possessing effective communication skills that allowed them to appropriately pace and tailor the information given and take the necessary time to explain the condition and answer parental questions. In terms of professional attributes, this included being perceived as a specialist, being credible, and working in an organization recognized as a center of excellence. The importance placed on knowledge and attributes of the person communicating the positive NBS result to families provides further support for the widespread use of specialist screening nurses who not only have knowledge of all conditions included in NBS but have also undergone relevant training related to breaking bad news and possibly even have counseling skills.

As previously reported [13,39], positive NBS results were associated with negative parental reactions, including feeling nausea, shock, disbelief, fear, and sadness. Previous research has reported the impact on parents [4,6,11] as well as on parent and child relationships [8] and family relationships [46,47]. This was reflected in the results of this study as parents talked about the impact on their relationship with the affected child, including being scared to bond with their child and the fear of being overprotective. In this study, the impact of the diagnosis on parental relationships ranged from bringing them closer together to causing a strain on the parental relationship. Parents also talked about the impact of sharing the news with family and friends; associated with this were feelings of responsibility, guilt, and a lack of understanding.

### Health Professionals’ Experiences of Delivering NBS Results

The experiences of health professionals delivering positive NBS results have been published elsewhere [1]. In summary, health professionals invested a lot of time and energy ensuring that the communication of positive NBS results to families was parent- and family-centered, but this could be influenced by the challenges they experienced, including inadequate information on the NBS card and parental reactions. As mentioned, a variety of methods for the delivery of positive NBS results have been reported previously [9,10,13,39-42] that are often determined by the seriousness of the condition. In this study, it became apparent that this was also to some extent dependent on local arrangements. The COVID-19 pandemic meant that telemedicine rapidly and unexpectedly became the medium for health consultations that had previously taken place face-to-face. Other research has indicated that staff found the use of telemedicine for the delivery of NBS results during the COVID-19 pandemic safe and effective [48], and recipients also considered it an acceptable alternative to face-to-face communication. Therefore, going forward, this may be an acceptable means of delivering positive NBS results to families that could be time-saving and, therefore, cost-effective if the content is well considered and the person delivering the result is knowledgeable about the relevant condition.

In addition to parental experiences, this study furthers our understanding of health professionals’ experiences with communicating positive NBS results to families. Health professionals involved in communicating positive NBS results are passionate about making sure that, although the message is distressing for parents, it is communicated well. Variations in communication practices continue to exist and are influenced by many factors, including the resources available and the lack of clear guidance. This affected not only the methods used to communicate positive NBS results but also the content of the communication to parents. This is supported by previous research conducted both nationally and internationally [4,6,41,49] suggesting that further guidance may be needed to ensure a more cohesive approach that meets the needs of parents and health professionals while being sensitive to the subtleties of each condition. However, the issue of finite resources and the need to prioritize them also requires careful consideration. Nevertheless, with clear evidence of the deleterious effects of poor communication practices on parents [4-12], this variability is neither reasonable nor conducive to building a positive rapport with families. This is vital to ensure concordance with treatment regimens and trust in health professionals to maximize outcomes for the children.

### Co-designed Interventions

To respond to the experiences and issues raised by parents and health professionals, EBCD, an established technique for gathering experiences and for co-design, was used [20-22,27,32,50-52]. It has been applied for the first time in this study to explore parents’ and health professionals’ experiences with the communication of positive NBS results. The process has enabled the prioritization of stakeholder requirements and the identification of co-designed solutions and additions to existing processes.

The co-designed interventions (changes to the NBS card; condition-specific, standardized laboratory proformas; condition-specific communication checklists; and an email or letter template to provide information to families following the communication of a positive NBS result) tackled different stages of the screening journey and areas where the participants felt that communication could be improved to minimize the anxiety and uncertainty experienced. These tools have been tailored to guide health professional communication with the aim of providing a more consistent experience. The interventions have subsequently been piloted at 2 sites; findings from this have been published elsewhere [15].

EBCD can be time-consuming and logistically challenging [27]; modifying the process has been shown to reduce costs [27]. The *Rethinking Strategies for Positive Newborn Bloodspot Screening Result Delivery: a process evaluation of co-designed interventions* project has been delivered during the COVID-19 pandemic; this has presented challenges in terms of bringing parents and health professionals together, a challenge that may continue for some time worldwide. We have adapted to these circumstances by using Basecamp as a collaborative tool enabling web-based EBCD outside the health care setting.

### Strengths and Limitations

This is the first known study that has explored communication pathways for positive NBS results from the laboratory to parents via clinical teams. Health professionals were recruited from clinical teams involved in managing all the conditions currently included in the NBS program. This increases the transferability of the study findings as previous work has mainly focused on CF and SCD. This is the only known study that has used EBCD to bring stakeholders together to develop co-designed interventions to improve the communication of positive NBS results.

In terms of limitations, health professionals were recruited via email; those with a pre-existing interest in this topic may have been more likely to self-select into the study. They may communicate results differently from providers who did not participate in the study, which may have biased the findings. However, health professionals were recruited from clinical teams involved in managing all the conditions currently included in the NBS program, which could have contributed to both the depth and breadth of the data collected. The researchers are experienced in this field, which may have biased data collection and analysis. Most parent participants were White British, which may limit the transferability of the findings.

### Implementation and Further Research

COVID-19 has meant that web-based consultations via platforms such as Microsoft Teams and Zoom are being used to communicate with families about their children’s positive NBS results. These have been described as an approximation to face-to-face interaction and are considered a visual upgrade of telephone consultations [53]. Initial studies that have explored these as a means of communicating positive NBS results to families suggest that they could be a safe and effective method for the delivery of positive NBS results to families [15,48]. Evidence suggests that video consultations (often referred to as telemedicine) have been viewed more favorably than telephone consultations [54]. The benefits of building rapport before using web-based approaches were found during teleconsultations in primary care during the lockdown [55]. The opportunities for using these web-based methods in NBS require further exploration to ensure that they are used appropriately, that the content of the message continues to be carefully crafted, and that the people involved are knowledgeable about the specific condition. However, a hybrid approach could act as a potential solution to address parental preferences, in particular face-to-face communication with their significant other present, communication via a condition-specific expert, and the clinical need for the timely provision of results.

In addition to the delivery of health care remotely, the pandemic has required web-based research and development. The adaptation of EBCD to include web-based methods could reduce costs while being easier to schedule. Adopting a web-based approach also has the potential to mitigate the imbalance of perceived power hierarchies [34] when patients and health professionals work together or, conversely, make it challenging to build a rapport. In this study, we benefited from the early stages of the process being run face-to-face, enabling relationships to develop. It is likely that a blended approach including face-to-face and web-based methods would help build effective relationships while offering flexibility and adaptation to the needs of parents (eg, childcare needs) and health professionals (eg, busy schedules). We argue that, as hybrid or blended ways of working are of increasing focus, the consideration and evaluation of different models of delivery for application in health care design would be beneficial.

### Conclusions

Staff involved in communicating positive NBS results are passionate about making sure that, although the message is distressing for parents, it is communicated well. Despite this, variations in communication practices continue to exist. This is influenced by many factors, including the resources available and the current lack of clear guidance. Parents and health professionals were able to successfully work together to share experiences, identify priorities, and develop potential solutions to improve the communication of positive NBS results to parents. The resulting co-designed interventions address communication at different stages of the communication pathway to improve the experiences of parents receiving positive NBS results for their children. Adopting a hybrid approach to EBCD that incorporates web-based co-design working groups could enhance the success of future EBCD projects.

## Abbreviations

CF: cystic fibrosis
EBCD: experience-based co-design
FST: family systems theory
MSUD: maple syrup urine disease
NBS: newborn bloodspot screening
NHS: National Health Service
PPI: Patient and Public Involvement
SCD: sickle cell disease
